# Oxidative stress and angiogenesis in primary hyperparathyroidism

**DOI:** 10.1007/s10353-016-0457-6

**Published:** 2016-12-14

**Authors:** Mariusz Deska, Ewa Romuk, Oliwia Anna Segiet, Grzegorz Buła, Witold Truchanowski, Dominika Stolecka, Ewa Birkner, Jacek Gawrychowski

**Affiliations:** 1 School of Medicine with the Division of Dentistry, Department of General and Endocrine Surgery, Medical University of Silesia, Bytom, Poland; 2 School of Medicine with the Division of Dentistry, Department of Biochemistry, Medical University of Silesia, Zabrze, Poland; 3 School of Medicine with the Division of Dentistry, Department of Histology and Embryology, Medical University of Silesia, Jordana 19, 41-808 Zabrze, Poland

**Keywords:** Primary hyperparathyroidism, Oxidative stress, Angiogenesis, Total antioxidant capacity, Total oxidative status

## Abstract

**Background:**

The inappropriate elevation of parathormone (PTH), which regulates the process of angiogenesis in parathyroid tissue, causes the changes of activity of enzymes responsible for the removal of free radicals. Parathyroidectomy (PTX) in patients with primary hyperparathyroidism (PHPT) lowers the level of PTH and leads to the reduction of risk of cardiovascular and all-cause mortality by normalization of the antioxidant status. Therefore, the aims of the study were to assess the activity of antioxidant enzymes and free radical reaction products in patients after parathyroidectomy, and to evaluate the correlation between the systemic oxidative stress and angiogenic parameters.

**Materials and methods:**

Patients with PHPT treated surgically were enrolled into the study. Total antioxidant capacity (TAC), total oxidative status (TOS), oxidative stress index (OSI), superoxide dismutase (SOD), ceruloplasmin (CER), lipid hydroperoxides (LHP) and malondialdehyde (MDA) were measured before and after parathyroidectomy. The immunohistological expression of angiogenic factors in parathyroid specimens was assessed by the BrightVision method from ImmunoLogic using murine monoclonal anti-human: anti-VEGF, anti-CD31 and anti-CD106 antibodies.

**Results:**

The significant increase of TAC, CER, reduction of TOS, MDA, SOD, especially for cytoplasmic form, and significant decrease of OSI, LHP were observed after PTX. There was no significant correlation between changes of oxidative stress markers and angiogenic parameters: VEGF, CD-31, CD-106 in parathyroid tissue. The correlation level was low and medium.

**Conclusions:**

Parathyroidectomy causes down-regulation of lipid peroxidation processes and leads to reduction of oxidative stress in patients with PHPT. The decrease in the OSI is the results of down-regulation of oxidative stress in the postoperative period. The change of the antioxidant status has no impact on angiogenesis processes in parathyroid tissue.

## Introduction

Primary hyperparathyroidism (PHPT) is caused by an inappropriate elevation of parathormone (PTH) level by one or more glands, which usually results in hypercalcemia [1]. Its prevalence has been estimated to be 0.1–0.3% in the general population and is more common in women than in men [2]. Excessive secretion of PTH directly stimulates bone resorption, increases calcium absorption in the intestine, inhibits the secretion of calcium by the kidney, indirectly enhances the production of vitamin D, which promotes calcium absorption in the intestine. Due to the results of studies revealing the impact of PHPT on the increase of the incidence of cardiovascular diseases, and probably also cancers, in patients with PHPT we should use surgical treatment [3–5]. Patients with primary hyperparathyroidism have increased risk of cardiovascular disease, which may be caused by a great number of factors, such as hypercalcemia [6], low vitamin D level [7], osteoporosis [8], or hyperphosphatemia [9]. Several studies demonstrated that oxidative stress also contributed to the development of vascular disorders [10]. Moreover, circulating oxidized plasma proteins, known as advanced oxidation protein products, play a role in the pathogenesis of chronic renal failure [11]. Endothelium is responsible for vascular tone, mainly due to the production of nitrogen monoxide (NO), which has been implicated in maintaining the balance between vasoconstriction and vasodilation. Vascular endothelial cells in diseases, including hypertension, hypercholesterolemia, and diabetes, produce increased amounts of reactive oxygen species (ROS), which cause formation of peroxynitrites and therefore neutralize NO activity. This leads to atherotrombosis and atherosclerosis [12]. Several reports suggested the possibility that parathyroidectomy could reduce the risk of cardiovascular complications [3].

The lack of calcium and phosphate homeostasis affects the functioning of the body’s defense mechanisms, eliminating free radicals, and thereby contributes to the abnormal cell oxidative profile [13]. The imbalance between the intensity of oxidation processes, which induce the formation of ROS, and antioxidative defense mechanisms is called oxidative stress. Parameters used to assess te oxidative stress are total oxidative status (TOS), total antioxidant capacity (TAC), and the oxidative stress index (OSI). Total oxidative status (TOS) is a parameter, which determines the total oxidant properties, whereas total antioxidant capacity (TAC) gives information about the antioxidant potential of body fluids in the organism, and is determined as the moles of oxidants neutralized by one liter of solution. OSI is defined as the ratio of the TOS level to TAC level [14]. One of the main consequences of ROS production is lipid oxidation, which has been implicated in the pathogenesis of many diseases. Lipids are oxidized in vivo by several different oxidants to give diverse products, in general lipid hydroperoxides (LHP) as the major primary product. One of the decay products is malondialdehyde (MDA), whose concentration increases due to enhanced production of ROS; therefore MDA is a lipid peroxidation marker used to assess lipid peroxidation caused by increased oxidative stress [15].

Reactive oxygen species can cause the oxidation of fats, proteins, DNA, and subsequently contribute to the damage of tissues. Toxic oxidation reaction products have cytostatic effect due to the cell membranes damage, and lead to cell death by apoptosis or necrosis. To avoid the mentioned consequences, in the body there are substances, acting as antioxidants, which inactivate and remove ROS. The balance is maintained by the antioxidant enzymes, such as superoxide dismutase, catalase, glutathione peroxidase, glutathione-S-transferase or non-enzymatic compounds, such as vitamins A, C, and E, bilirubin, albumin, uric acid, and ceruloplasmin. Ceruloplasmin (CER) is an antioxidant, which is responsible for scavenging ROS such as singlet, superoxide, and hydroxyl radicals. This glycoprotein has been implicated in copper transport, iron turnover, ferroxidase, glutathione peroxidase, and ascorbate oxidase activities. Its expression has been demonstrated in the liver, spleen, lung, testis, and brain [16]. Sulfhydryl (SH) groups, also known as thiol or sulphur groups, play a huge role in destruction of free peroxidized fatty acids and hydrogen peroxide. Glutathione (GSH) is a tripeptide with SH group, and a cosubstrate for the peroxidation inhibiting protein, which causes decomposition of hydroperoxide fatty acids located in phospholipids [17]. Sulfhydryl groups are particularly susceptible to the effects of free radicals. Several researches indicated that the assessment of the thiol groups was a better indicator of oxidative stress than the measurement of the total oxidative status (TOS) or total antioxidant capacity (TAC) [18]. In most biological processes, superoxide anion is the first generated ROS, which results in the formation of subsequent ROS. Superoxide dismutase (SOD) is an ubiquitous enzyme, constituting the first line of defense, which catalyzes the conversion of superoxide anion into hydrogen peroxide and oxygen. There are four families of SOD, defined by their metal ion binding partners: Fe, Mn, Ni, or both Cu and Zn. CuZnSOD is a copper and zinc-containing homodimer that is found almost exclusively in intracellular cytoplasmic spaces. Mn-SOD exists as a tetramer and is initially synthesized with a leader peptide, which targets this manganese-containing enzyme exclusively to the mitochondrial spaces. EC-SOD is the most recently characterized SOD, exists as a copper and zinc-containing tetramer, and is synthesized with a signal peptide that directs this enzyme to extracellular spaces [19]. The enzymes decomposing hydrogen peroxide are catalase (CT) [20] and glutathione peroxidase (GPx) [21]. Another role of GPx in defense against ROS is to catalyze the coupling reaction of products of lipid peroxidation with glutathione aldehyde. By measuring the activity of the antioxidant system we can indirectly measure free-radical activity [21].

ROS are also involved in the transmission of signals, cell differentiation, and apoptosis. They may affect the functioning of the mechanism of new cells and blood vessels formation [22]. Hypoxia is a known factor up-regulating the production of free radicals, which is also a stimulator of angiogenesis [23]. This is a multistep process of formation of new blood vessels from the pre-existing microvasculature, during which endothelial cells migrate and proliferate [23]. Physiologically, it is essential for proper development, growth, and maturation of the human body and is controlled by a balance between pro- and anti-angiogenic factors and their receptors. Neovascularization is also implicated in the pathogenesis of many diseases, particularly neoplasia [24]. Because the endocrine glands are well vascularized and the structure of the vessel facilitates the exchange of various substances, including hormones, they are a common experimental model in the study on angiogenesis. Parathyroid tissue can promote spontaneous induction of angiogenesis in vitro [25] and in vivo models in a vascular endothelial growth factor-dependent manner [26]. Autotransplanted parathyroid tissue after thyroidectomy is able to produce new blood vessels and synthesize parathormone, which is responsible for maintaining calcium homeostasis [27]. Several studies demonstrated that also cultured parathyroid cells could trigger angiogenesis [26]. The strongest regulator of normal and pathological angiogenesis is vascular endothelial growth factor (VEGF). Vascular endothelial growth factor, also known as vascular permeability factor (VPF), plays a huge role in angiogenesis during embryogenesis, skeletal growth, and pathological angiogenesis associated with tumor development. Local hypoxia up-regulates VEGF synthesis [28]. Several studies confirmed its contribution to the pathogenesis of diseases, such as breast cancer, renal cell carcinoma, colorectal cancer, angiosarcoma, rheumatoid arthritis, and diabetic retinopathy [29–31], Moreover, PTH regulates the expression of VEGF and metalloproteinases (MMPs) [32, 33].

Platelet endothelial cell adhesion molecule (PECAM-1), denoted cluster of differentiation 31 (CD31), is a member of type I integral membrane glycoprotein family. It is expressed on endothelial cells, peripheral lymphoid cells, platelets, monocytes, and neutrophils. CD 31 is implicated in angiogenesis, leukocyte migration, T cell activation, platelet aggregation, regulation of endothelial junctional integrity, and integrin activation [34, 35].

Vascular cell adhesion molecule-1 (VCAM-1, CD106) is present on activated vascular endothelial cells, follicular and interfollicular dentritic cells of lymph nodes, macrophages myoblasts, and bone marrow stromal cells. CD106 regulates the adhesion of lymphocytes, monocytes, eosinophils, and basophils to vascular endothelium through interaction with integrin receptors. It is also responsible for transmigration and co-stimulation of T cell proliferation [36].

The activation mechanism of angiogenesis and oxidative stress is still not completely known, but probably it remains under control because most of parathyroid tumors are benign and do not have the ability to metastasize [1, 2]. What is more, of interest is the impact of systemic oxidative stress occurring in the PHPT on the severity of angiogenesis in parathyroid tissue.

Therefore, the aims of the study were the following:

1. Assess the activity of antioxidant enzymes and free radical reaction products in patients after parathyroidectomy;
2. Evaluate the correlation between the systemic oxidative stress and angiogenic parameters.

## Materials and methods

Blood serum and parathyroid tissue specimens, collected from 56 consecutive patients with PHPT treated surgically (age 55.66 ± 14.22 years), constituted our study material (Table 1). Blood samples for biochemical examinations were collected from patients 24 h before the surgery, as well as on postoperative day 15. At each time point, 5 ml of blood were aspirated into both vacuum tubes and vacuum tubes with an anticoagulant (EDTA). In order to obtain a hemolysate, 0.9% NaCl was added to the remaining blood cells. The material was mixed and centrifuged for 10 min at 3000 revolutions/min. Supernatant was removed and again 0.9% NaCl was added. The procedure was repeated twice. Afterward, 0.4 ml of blood was collected from the bottom of the test tube and 3.6 ml of doubly distilled water was added. After mixing, we obtained 10% blood cell lysate. The material was frozen at −80 °C. After thawing, biochemical examinations were performed.

**Table 1:** Group characteristics

Study group		*N* = 56
Mean age in years		55.6 (28–82)
Sex	Male	11 (19.6%)
	Female	45 (81.4%)
Histopathology	Adenomas	36 (64.3%)
	Hyperplasia	17 (30.35%)
	Carcinoma	3 (5.35%)
Type of surgery	Open parathyroidectomy	56 (100%)

### Biochemical analysis

#### Analysis of TOS and TAC

The TOS and TAC were determined in serum by the method described by Erel. TOS measurements were done by reading at end-point 560 nm in the spectrophotometer 3–4 min after mixing the samples and reagents, and the results were expressed in hydrogen peroxide liter (µmol H_2_O_2_ equiv/l). The method is based on the oxidation of ferrous ion to ferric ion in the presence of various oxidant species in acidic medium and the measurement of the ferric ion by xylenol orange [37]. TAC was assessed by the use of a standard antioxidant solution called Trolox equivalent which is analogous to vitamin E. TAC was presented as mmol Trolox equiv/l. TAC measurements were performed by kinetic reading in the spectrophotometer 5 min after the sample and reagent were mixed [38].

#### Analysis of OSI

After TAC and TOS measurements, the OSI levels, which allow us to make an exact comment on the oxidant and antioxidant balance, were calculated according to the following formula specified in the catalog of the kit (rel assay diagnostics): OSI = (TOS µmol/l)/(TAC [mmol Trolox equiv/l] × 100).

#### Analysis of antioxidants

The method of Oyanagui was used to measure the activity of superoxide dismutase (SOD) and its isoenzymes: the cytoplasmic Cu/Zn-superoxide dismutase (Cu/ZnSOD) and the mitochondrial Mn-superoxide dismutase (MnSOD). In this method, xanthine oxidase produces superoxide anions, which react with hydroxylamine forming nitric ions. This ions react with naphthalene diamine and sulfanilic acid, generating a colored product. Concentration of this product is proportional to the amount of produced superoxide anions and negatively proportional to the activity of SOD. The enzymatic activity of SOD was expressed in nitric units. The activity of SOD is equal to 1 nitric unit (NU) when it inhibits nitric ion production by 50%. Activities of SOD were normalized to milligram of protein in homogenates (NU/mg protein) [39]. The concentration of sulfhydryl groups (SH) in serum was determined by Koster method using 5,5’-dithiobis(2-nitrobenzoic acid)—DTNB. Concentration was shown in mmol/l [40]. Serum ceruloplasmin was determined spectrophotometrically using the Richterich reaction with *p*-phenyldiamine [41].

#### Analysis of lipid oxidation products

Lipid hydroperoxide concentration in serum was determined by Södergren et al. using xylene orange. Values were expressed in mmol/l. All this parameters were measured with the use of the Perkin Elmer spectrophotometer Victor X3 [42]. The malondialdehyde level was determined by the Ohkawa method using a Perkin Elmer LS45 spectrofluorometer. The concentration of MDA was expressed as mol/g of protein [43].

### Immunohistochemical analysis

For immunohistology, all specimens were immediately fixed for 20 min in cold acetone (−20 °C) and immersed in embedding medium (OCT Compound, Miles Inc.), and all of them were cut serially into 5 μm thickness slides, air dried at room temperature, and assayed. Frozen sections were incubated with murine monoclonal anti-human: anti-VEGF (clone SP28), anti-CD31 (clone JC/70A), and anti-CD106 (clone 1.4C3). The dilution of the primary antibodies was 1:500 and was verified in a series of pilot experiments. The immunohistological investigations were performed by the BrightVision method from ImmunoLogic, according to the manufacturer’s instructions. The primary antibody was omitted from negative control slides. The sections were counterstained with Mayer’s hematoxylin. Each specimen was evaluated qualitatively, semiquantitatively (score index from 0 to 3+), and quantitatively. Semiquantitative score index was as follows: (0): no staining; (1+): weak focal staining; (2+): multifocal moderate staining; and (3+): diffuse strong staining. Positively stained cells were counted in all cryostat sections in at least 10 high power fields (HPF) per each biopsy under 400x magnification and averaged for each field using Nikon Eclipse 80i microscope with DSFi1 digital camera and NIS Elements software form Nikon.

All patients gave their informed consent. The protocol was approved by the institutional ethics committee.

### Statistical analysis

All statistical analyses were done with the use of STATISTICA 10 program. The normality of the results distribution was verified using the Shapiro–Wilk test. Due to the small size of the groups we used non-parametric “U” Mann–Whitney test, and the data were presented as median with the first and fourth quartiles. The results were considered statistically significant if *p* < 0.05. The lack of statistical significance was presented as NS (nonsignificant).

## Results

### The activity of antioxidant enzymes and free radical reaction products in patients before and after parathyroidectomy

**Fig. 1:**
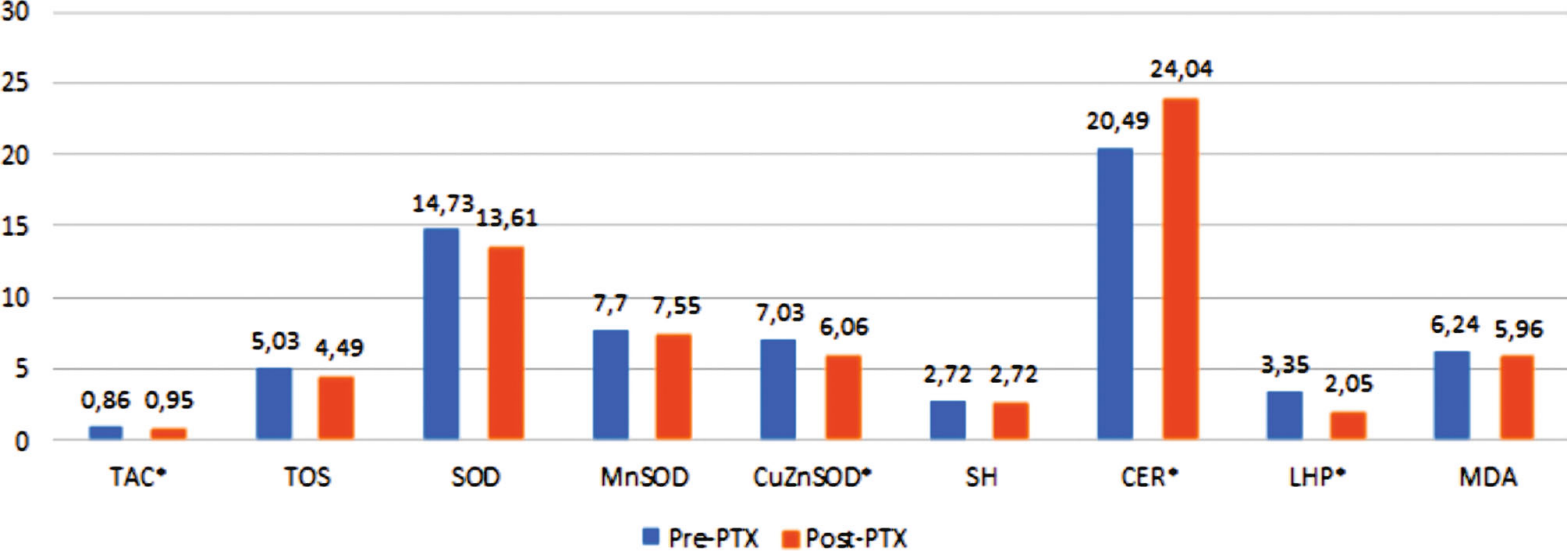
The changes of serum oxidative stress index, serum level of antioxidants, and serum lipid peroxidase products between groups before and after parathyroidectomy. Mann–Whitney U‑test was applied. The data were presented as median (minimum–maximum) unless stated otherwise. ^*^Significant *P*-value (*P* < 0.05). *pre-PTX* before parathyroidectomy, *post-PTX* 15 days after parathyroidectomy, *TAC* total antioxidant capacity, *TOS* total oxidant status, *SOD* superoxide dismutase, *MnSOD* mitochondrial form SOD, *CuZnSOD* cytoplasmic form SOD, *SH* sulfhydryl groups, *CER* ceruloplasmin, *LHP* lipid hydroperoxides, *MDA* malondialdehyde

Parathyroidectomy in patients with PHPT resulted in the significant increase of TAC (*p* < 0.001) and substantial reduction of TOS (*p* = 0.027). Moreover, a considerable decrease in the oxidative stress index (OSI) was demonstrated (*p* < 0.05), which was the results of down-regulation of oxidative stress in postoperative period (Table 2). Among measured antioxidants, we observed the significant decrease in serum level of SOD (*p* < 0.05) mainly in cytoplasmic form CuZnSOD (*p* < 0.05) and the significant increase of ceruloplasmin (*p* < 0.05). The serum concentration of SH remained unchanged after parathyroidectomy. After surgery, we observed a significant decrease of lipid hydroperoxides (*p* < 0.05) and a decrease of malonic dialdehyde. Both were caused by down-regulation of ROS (Table 2). The results are presented in Fig. 1.

**Table 2:** The comparison of serum oxidative stress index, serum level of antioxidants, and serum lipid peroxidase products between groups before and after parathyroidectomy. Parathyroidectomy (*PTX*) in patients with PHPT resulted in the significant increase of TAC (*p* < 0.001) and substantial reduction of TOS (*p* = 0.027). Moreover, a considerable decrease in the oxidative stress index (*OSI*) was demonstrated (*p* < 0.05), which was the results of down-regulation of oxidative stress in postoperative period. A significant decrease in serum level of SOD (*p* < 0.05) mainly in cytoplasmic form CuZnSOD (*p* < 0.05) and the significant increase of ceruloplasmin (*p* < 0.05) were observed. The serum concentration of SH remained unchanged after parathyroidectomy. After surgery a significant decrease of lipid hydroperoxide (*LHP*, *p* < 0.05) and a decrease of malonic dialdehyde (*MDA*) were demonstrated. Both were caused by down-regulation of ROS

Measured parameters	Pre-PTX group	Post-PTX group
TAC mmol Trolox equiv/l	0.86 ± 0.15	0.95 ± 0.14^*^
TOS µmol H_2_O_2_ Equiv/L	5.03 ± 1.84	4.49 ± 1.76
OSI AU	584.88	472.62^*^
SOD NU/ml	14.73 (9.63–20.25)	13.61 (7.4–20.1)
MnSOD NU/ml	7.70 (2.58–16.42)	7.55 (2.51–15.65)
CuZnSOD NU/mg	7.03 (2.9–10.88)	6.06 (0.74–11.83)^*^
SH umol/g protein	2.72 (0.97–3.79)	2.72 (1.01–4.00)
CER mg/dl	20.49 (5.56–43.22)	24.04 (9.47–58.37)^*^
LHP mmol/l	3.35 ± 1.83	2.05 ± 1.98^*^
MDA mol/g of protein	6.24 ± 1.58	5.96 ± 1.54

Mann–Whitney *U*-test was applied. The data were presented as median (minimum–maximum) unless stated otherwise

*pre-PTX* before parathyroidectomy, *post-PTX* 15 days after parathyroidectomy, *TAC* total antioxidant capacity, *TOS* total oxidant status, *OSI* oxidative stress index, *SOD* superoxide dismutase, *MnSOD* mitochondrial form SOD, *CuZnSOD* cytoplasmic form SOD, *SH* sulfhydryl groups, *CER* ceruloplasmin, *LHP* lipid hydroperoxides, *MDA* malondialdehyde

^*^Significant *P*-value (*P* < 0.05)

### The correlation of angiogenesis in the parathyroid tissue with the parameters of oxidative stress

The expression of angiogenic factors: VEGF, CD31, CD106 in parathyroid tissue was negatively correlated with the changes of the TOS, TAC, SOD, and LHP but positively correlated with MDA. There was no statistical significance in the study group patients (Table 3). The correlation level was low and medium. Among the studied biochemical parameters, the correlation between the expression of angiogenic factors with serum parathyroid hormone is noteworthy (Table 4).

**Table 3:** The correlation between stress markers and angiogenic factors in parathyroid tissue in patients with PHPT. No significant relationship between the angiogenesis and systemic changes in oxidative stress in patients operated due to primary hyperparathyroidism was demonstrated

Stress markers correlation factor r		TOS	TAC	SOD	LHP	MDA
VEGF+	pre-PTX	0.22	0.44	0.05	−0.12	0.13
	post-PTX	−0.23	−0.17	−0.05	−0.28	0.24
CD31+	pre-PTX	0.29	0.44	0.12	−0.02	0.15
	post-PTX	−0.42	−0.05	−0.08	−0.37	0.22
CD106+	pre-PTX	0.11	0.33	0.13	−0.03	0.03
	post-PTX	−0.39	0.06	0.17	−0.36	0.02

Mann–Whitney *U*-test was applied

*pre-PTX* before parathyroidectomy, *post-PTX* 15 days after parathyroidectomy, *TAC* total antioxidant capacity, *TOS* total oxidant status, *SOD* superoxide dismutase, *LHP* lipid hydroperoxides, *MDA* malondialdehyde, *VEGF* vascular endothelial growth factor, *CD31* platelet endothelial cell adhesion molecule, *CD106* vascular cell adhesion molecule-1

**Table 4:** The correlation between the changes of serum PTH level and angiogenic parameters in parathyroid tissue. A strong correlation between PTH level and the expression of all angiogenesis markers, including VEGF, CD31, and CD106, was revealed, which proves that parathormone is implicated in angiogenesis in the parathyroid glands

	PTH pre-PTX 544.21 µmol/l ± 793.77	PTH post-PTX 27.90 µmol/l ± 32.34
VEGF+	r = 0.65^*^	r = 0.67^*^
CD31+	r = 0.52^*^	r = 0.36
CD106+	r = 0.50^*^	r = 0.56^*^

Mann–Whitney *U*-test was applied

*pre-PTX* before parathyroidectomy, *post-PTX* 15 days after parathyroidectomy, *VEGF* vascular endothelial growth factor, *CD31* platelet endothelial cell adhesion molecule, *CD106* vascular cell adhesion molecule-1

^*^Significant *P*-value (*P* < 0.05)

## Discussion

Our study demonstrated decreased oxidative stress markers after parathyroidectomy, which may be one of the reasons of decreased probability of cardiovascular events and a survival benefit. In the postoperative period in patients with primary hyperparathyroidism, a significant increase of the total antioxidant capacity and a reduction of total oxidative status were revealed. Moreover, a considerable decrease in the oxidative stress index was demonstrated, which was the result of down-regulation of oxidative stress in postoperative period. Among measured antioxidants, we observed a significant decrease in serum level of SOD, mainly in cytoplasmic form CuZnSOD, which was due to decrease in production of superoxide anion, associated with the normalization of parathormone levels, and the significant increase of ceruloplasmin. Malondialdehyde concentration in the blood of patients with primary hyperparathyroidism was reduced, which was probably triggered by the inhibition of lipid peroxidation. These data confirmed the previous study by Tanaka et al., which showed the case of primary hyperparathyroidism, in which oxidative stress was significantly lower after parathyroidectomy [44]. The results suggest that parathyroidectomy reduces oxidative stress in patients with primary hyperparathyroidism, which may in part explain reduced risk of cardiovascular and all-cause mortality after parathyroidectomy.

Nevertheless, the reason for decreased oxidative stress after parathyroidectomy is still not completely known. We could suspect that the surgery could cause the changes in oxidative status; however, the surgery itself is a widely known factor triggering ROS production [45, 46]. What is more, surgical trauma is responsible for metastasis development and promotion of tumor growth postoperatively, and this complication is related to ROS-rich environment in the postoperative period [47, 48]. On the other hand, Kaçmaz et al. revealed that nitric oxide and malondialdehyde levels were enhanced and catalase levels were decreased in patients after both thyroidectomy and thyroparathyroidectomy [49].

Another factor involved in changes in oxidative stress could be the level of calcium (Ca). The excessive accumulation of Ca in mitochondria induces membrane permeability transition, release of cytochrome c, respiratory inhibition, and ROS production. The mitochondrial Ca overload also results in oxidative stress in cardiomyocytes after ischemia-reperfusion. Ca overload and oxidative and nitrosative stress in peripheral blood mononuclear cell (PBMC) lead to higher production of hydrogen dioxide (H_2_O_2_) and in turn take part in transmitting signals activating PBMC. Previous parathyroidectomy inhibits ROS synthesis [13].

Several studies suggested that parathormone could be the factor implicated in oxidative stress imbalance and causally involved in pathological processes leading to cardiovascular disease. After parathyroidectomy, serum PTH decreases rapidly to reach physiological values. Higher plasma PTH was associated with higher risk for cardiovascular mortality independently of established cardiovascular risk factors and factors associated with mineral homeostasis [50, 51]. Sambrook et al. found that serum PTH was correlated with increased mortality in the frail elderly, regardless renal parameters, vitamin D levels, and bone mass assessed by ultrasound examinations [52]. Higher PTH levels were associated with increased mortality and severity of illness in critically ill patients in an emergency or intensive care department, independently of serum calcium levels. Increased PTH concentration was also correlated with enhanced fatty acid oxidation in the myocardium in animals with secondary hyperparathyroidism caused by chronic renal failure [53]. In accordance with this research are the results of experimental studies which revealed PTH receptors in vascular endothelial cells, suggesting that the endothelium is a target organ of PTH [54, 55].

There are several possible explanations for the impact of PTH on cardiovascular mortality. PTH is responsible for regulating the level of ionized calcium via stimulation of osteoclastic bone resorption, stimulation of calcium reabsorption in the distal tubule of the kidney, and activation of 25-hydroxyvitamin D 1‑alpha hydroxylase in the proximal renal tubule. The imbalance in mineral homeostasis leads to cardiovascular complications. PTH is also involved in vascular calcification and remodeling and therefore causes atherogenesis [56–58]. Excessive levels of PTH result in ventricular hypertrophy, cardiac calcification, and fibrosis [59–61]. Moreover, up-regulated PTH concentrations have been implicated in inflammation, raised systolic blood pressure, and increased body mass index [4, 62–64]. Therefore, the impact of parathormone on oxidative stress in patients with primary hyperparathyroidism may also be one of the factors implicated in the pathogenesis of cardiovascular complications in primary HPT.

Oxidative stress also plays a huge role in the process of angiogenesis. This process is necessary when a tumor reaches a critical volume, which is approximately 1–2 mm^3^, in order to continue its expansion [65, 66]. Nicotinamide adenine dinucleotide phosphate oxidases (Nox)-derived ROS and VEGF promote the angiogenic switch in cancer cells [67, 68]. Nox-derived ROS play a role not only in pathological states, but also via H_2_O_2_ synthesis contribute to the processes of angiogenesis and tissue repair [69–72]. Reactive oxygen species stimulate the activation of hypoxia-inducible factor 1‑alpha (HIF-1α) and thus enhance VEGF expression. When VEGF binds to VEGF receptor 2, it triggers ROS release, which is implicated in basement membrane degradation, migration, proliferation, and vessel formation [68, 73, 74].

In our study no significant relationship between the angiogenesis and systemic changes in oxidative stress in patients who underwent surgery due to primary hyperparathyroidism was demonstrated. There was no significant correlation between the changes in both the level of antioxidants, as well as lipid peroxidation products and the expression of angiogenic markers. Nevertheless, endothelial cells are highly heterogeneous and it is suggested that the panendothelial cell markers are not ideal indicators of pathological or activated neovessels. However, there was a strong correlation between PTH level and the expression of all angiogenesis markers, including VEGF, CD31, and CD106, which proves that parathormone is implicated in angiogenesis in the parathyroid glands.

In conclusion, this study showed that parathyroidectomy was a procedure of a great importance to reduce oxidation processes in patients with PHPT. The reduction of the level of parathormone in the postoperative period plays a crucial role in the down-regulation of free radical production and lowers the mortality of patients with PHPT from cardiovascular diseases. It has not been established what time is required to obtain a complete normalization of blood parameters. The mechanism of oxidative stress in the parathyroid gland is a complex process depending on many factors, among which of great importance is PTH. The phenomenon of angiogenesis in the parathyroid glands is also associated with parathormone, due to the strong correlation between these parameters. Even if a great number of studies have been performed recently, the mechanisms implicated in the pathogenesis of primary hyperparathyroidism and processes occurring after surgical resection are still not completely known. A better understanding of these issues could result in more precise assessment of diagnosis and more effective treatment, especially in those cases, in which commonly used parameters are insufficient. Studies of this type should be continued and new insight may in the future result in targeted therapy or possibly prevention.
